# Targeting innate immune sensors for therapeutic strategies in infectious diseases

**DOI:** 10.71150/jm.2503009

**Published:** 2025-06-30

**Authors:** Seyun Shin, Young Ki Choi, SangJoon Lee

**Affiliations:** 1 Department of Biological Science, Ulsan National Institute of Science and Technology (UNIST), Ulsan 44919, Republic of Korea; 2 Center of Study of Emerging and Re-emerging Viruses, Korea Virus Research Institute, Institute for Basic Science (IBS), Daejeon 34126, Republic of Korea; 3 Graduate School of Health Science and Technology, Ulsan National institute of Science and Technology (UNIST), Ulsan 44919, Republic of Korea

**Keywords:** innate immunity, pathogen, infection, inflammation, cell death

## Abstract

The innate immune system relies on innate immune sensors, such as pattern recognition receptors (PRRs), to detect pathogens and initiate immune responses, crucial for controlling infections but also implicated in inflammatory diseases. These innate immune sensors, including Toll-like receptors (TLRs), nod-like receptors (NLRs), RIG-I-like receptors (RLRs), absent in melanoma 2 (AIM2), and Z-DNA binding protein 1 (ZBP1) trigger signaling pathways that produce cytokines, modulating inflammation and cell death. Traditional therapies focus on directly targeting pathogens; however, host-targeting therapeutic strategies have emerged as innovative approaches to modulate innate immune sensor activity. These strategies aim to fine-tune the immune response, either enhancing antiviral defenses or mitigating hyperinflammation to prevent tissue damage. This review explores innate immune sensor-based therapeutic approaches, including inhibitors, agonists, and antagonists, that enhance antiviral defense or suppress harmful inflammation, highlighting innate immune sensors as promising targets in infectious and inflammatory disease treatment.

## Introduction

Innate immunity serves as the body first line of defense against pathogens, including viruses, bacteria, and parasites. This rapid response is initiated immediately or within hours of encountering pathogens (Marshall et al., 2018), and it is driven by pattern recognition receptors (PRRs) that detect pathogen-associated molecular patterns (PAMPs) and damage-associated molecular patterns (DAMPs) such as HMGB1, ATP, and uric acid.

**Fig. 1.**
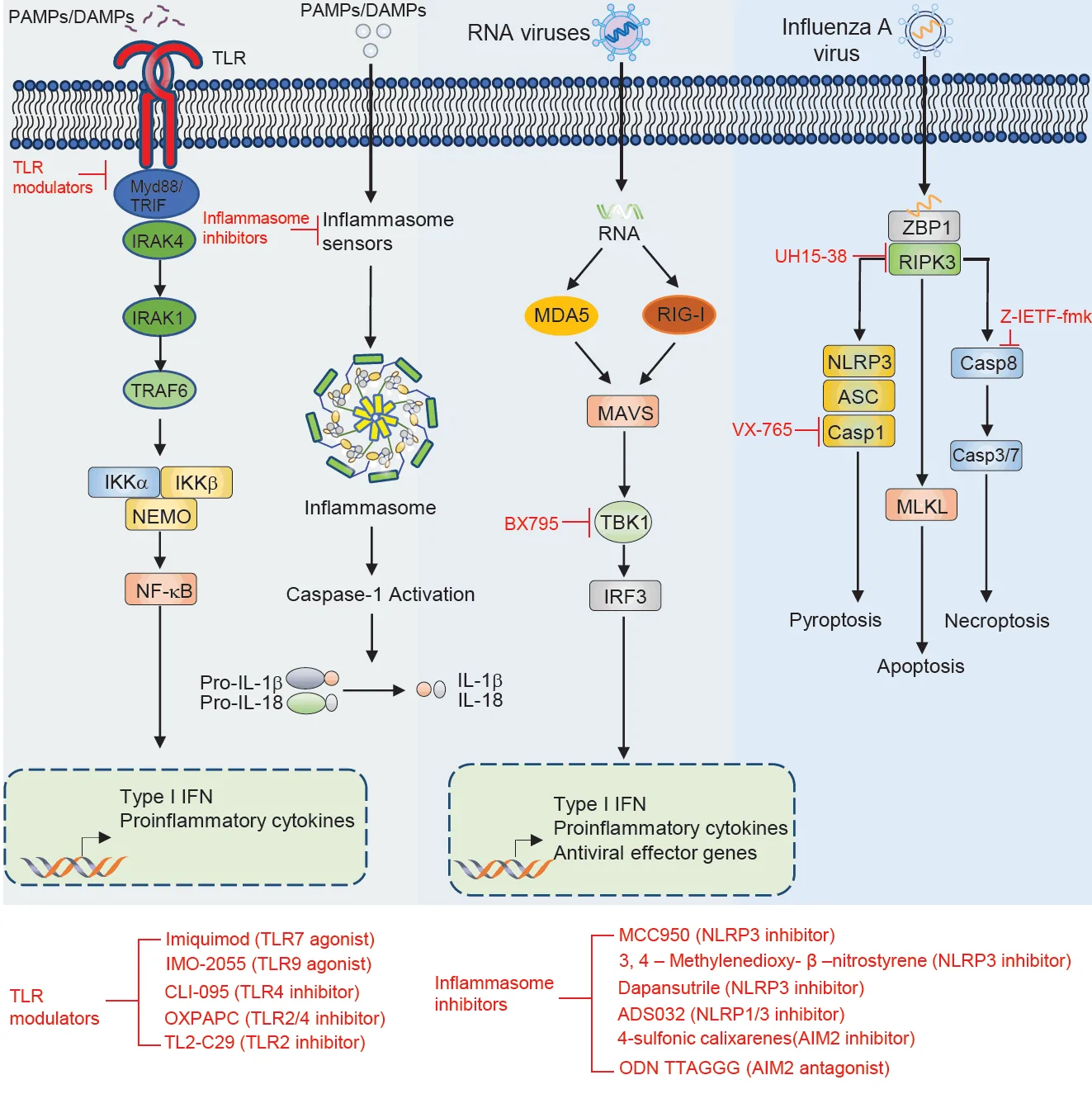
Innate immune sensors response to various stimuli. Toll-like receptors (TLRs) detect ligands such as lipopolysaccharide (LPS), zymosan, flagellin, double-stranded RNA (dsRNA), and CpG DNA, signaling through Myeloid Differentiation Primary Response 88 (MyD88)- or TIR-domain-containing Adapter-inducing Interferon-β (TRIF)-dependent pathways to induce inflammatory cytokines. Endosomal TLRs, including TLR3, TLR7, TLR8, and TLR9, recognize viral or endogenous nucleic acids. Canonical inflammasomes, assembled by Nod-like receptors (NLRs) such as NOD-like Receptor Family Pyrin Domain-containing 1 (NLRP1), NLRP3, and NLR containing CARD 4 (NLRC4), as well as Absent in Melanoma 2 (AIM2) and pyrin, recruit pro-caspase-1 via the Apoptosis-associated Speck-like Protein Containing a CARD (ASC) adaptor. This activates caspase-1, promoting the release of interleukin-1β (IL-1β) and IL-18. NLRP3 responds to bacterial toxins, viral RNA, ATP, and reactive oxygen species (ROS). The RIG-I-like receptor (RLR) family, including Retinoic Acid-Inducible Gene I (RIG-I), Melanoma Differentiation-Associated Gene 5 (MDA5), and Laboratory of Genetics and Physiology 2 (LGP2), detects viral RNA. Z-DNA-Binding Protein 1 (ZBP1) recognizes Influenza A virus (IAV) and induces pyroptosis, apoptosis, or necroptosis.

PAMPs are highly conserved molecular patterns that are specific to and commonly found in certain types of pathogenic microorganisms. Innate immune cells recognize PAMPs through PRRs and distinguish “self” and “non-self”. When the host is stimulated by tissue damage, cell necrosis, or other factors, it produces certain proteins and metabolites (Gong et al., 2020), these molecules are known as DAMPs. PRRs, such as Toll-like receptors (TLRs), nucleotide-binding oligomerization domain (NOD)-like receptor family proteins (NLRs), retinoic acid-inducible gene I (RIG-I)-like receptors (RLRs), absent in melanoma 2 (AIM2), and Z-DNA binding protein 1 (ZBP1)(Enzan et al., 2023), play a crucial role in activating the innate immune system by detecting PAMPs and DAMPs. Each PRR plays an essential role in sensing specific ligands and initiating signaling pathways that regulate gene expression, protein synthesis, release of cytokine and chemokine, and cell death (Kanneganti, 2020) (Fig. 1).

In this review, we discuss how innate immune sensor detect pathogens through PRRs and explore current therapeutic strategies that target innate immune sensors.

## Toll-Like Receptors (TLRs)

Toll-like receptors (TLRs) are one of the first identified PRRs and are pivotal in initiating inflammatory responses (Fitzgerald and Kagan, 2020). TLRs are membrane-bound signal receptors and TLRs have dual functions: binding specifically to their ligands and transmitting signal to amplify the effect of anti-pathogen infection (Li and Wu, 2021). In humans, TLR1 to TLR10 have been found, whereas in mice, TLR1 to TLR9, and TLR11 to TLR13 are present, while TLR10 is not functional due to the insertion of reverse transcriptase (Balachandran et al., 2022). The recognition of PAMPs by TLRs depends on their cellular localization, which dictates the types of ligands they recognize and the mechanisms of recognition. Certain TLRs are found on the surface of immune cells, either as homodimers or heterodimers, and primarily detect membrane components of pathogens, including lipids, lipoproteins, and proteins. In contrast, other TLRs, such as TLR3, 7, 8, and 9 are expressed intracellularly as homodimers and are specialized in recognizing microbial nucleic acids (Chuenchor et al., 2014; Li and Wu, 2021).

TLR1 and TLR6 can combine with TLR2 to form TLR1/TLR2 or TLR6/TLR2 heterodimers, enabling the recognition of tri-acylated lipopeptides and di-acylated lipopeptides (Farhat et al., 2008). Several PAMPs can stimulate TLR4, these molecules include lipopolysaccharide (LPS) from Gram-negative bacteria, the fusion (F) protein of respiratory syncytial virus (RSV) and the envelope protein of mouse mammary tumor virus (MMTV) (Kurt-Jones et al., 2000; Lu et al., 2008; Rassa et al., 2002). Flagellin binding to TLR5 leads to the activation of MyD88-dependent signaling pathways (Yoon et al., 2012). This process triggers the proinflammatory transcription factor nuclear factor kappa-light-chain-enhancer of activated B cells (NF-kB) in various cells, including epithelial cells, monocytes, and dendritic cells, thereby initiating innate immune responses against bacteria with flagella (Eaves-Pyles et al., 2001; Gewirtz et al., 2001; Hayashi et al., 2001; McDermott et al., 2000; Means et al., 2003). TLR3 is essential for identifying double-stranded RNA (dsRNA), a key factor in the detection of viral infections (Chattopadhyay and Sen, 2014). TLR7 is known to detect guanosine- or uridine-rich single stranded RNA (ssRNA) from various viruses such as human immunodeficiency virus (HIV), vesicular stomatitis virus (VSV), and influenza virus (Diebold et al., 2004). TLR8 is closely related to TLR7, and both genes are located on the X chromosome. TLR9 is crucial for recognizing the CpG motif of bacterial and viral DNA, as TLR9-deficient mice fail to respond to CpG DNA (Hemmi et al., 2000; Takeda and Akira, 2015).

TLRs are key membrane bound PRRs that detect PAMPs and initiate innate immune responses by recognizing a diverse range of ligands based on their cellular localization and receptor specificity.

## Nod-Like Receptors

The nucleotide oligomerization domain (Nod)-like receptors (NLRs) are intracellular cytosolic sensors (Franchi et al., 2009). NLRs are crucial for detecting molecules related to intracellular infections (Yu et al., 2024). Some NLRs such as NLRP1, NLRP3, and NLRC4 are specialized in triggering the activation of an intracellular complex known as inflammasomes (Almeida-da-Silva et al., 2023). NLRP6 and NLRP9b have broader physiological functions, including gut homeostasis and antiviral defense (Venuprasad and Theiss, 2021; Zhu et al., 2017). Inflammasomes are multi-protein complexes that assemble within the host cell in response to PAMPs or different forms of stress can be released into the extracellular space, where they contribute to inflammation (Broz and Dixit, 2016; Lee et al., 2021a; Martinon et al., 2002).

Few ligands have been found for NLRP1 to date, which include bacterial products such as lethal toxin (LT) produced by *Bacillus antharacis* which activates murine NLRP1b (Levinsohn et al., 2012), muramyl dipeptide (MDP) (Zhong et al., 2013), a component of bacterial peptidoglycan that activates human NLRP1(Feldmeyer et al., 2007). The murine NLRP1b inflammasome is activated by a reduction in cytosolic ATP levels (Chavarria-Smith and Vance, 2013; Frew et al., 2012; Hellmich et al., 2012; Liao and Mogridge, 2013). NLRP3 plays a role primarily in the formation of an inflammasome complex and NLRP3 inflammasome is the most well-known inflammasome. The activation of the NLRP3 inflammasome requires two signals: first, a priming signal, triggered by PAMPs such as LPS, activates the NF-kB pathway and consequent upregulation of NLRP3, pro-interleukin-1beta (IL-1β) and pro-IL-18; and second, an activation signal, which is provided by various stimuli, such as DAMPs. Many stimuli can activate the NLRP3 inflammasome, such as extracellular ATP, ROS generation, mitochondrial dysfunction, viral infection (Almeida-da-Silva et al., 2023; Lee et al., 2019).

The NLR family of apoptosis inhibitory proteins (NAIPs) represents a well-characterized NLR sub-family. NAIP proteins function as specific cytosolic receptors for various bacterial protein ligands. NAIPs assemble with a downstream protein NLRC4, interferon regulatory factor 8 (IRF8) (Karki et al., 2018) is required to form an NLRC4 inflammasome (Vance, 2015). NAIP5 in mice recognizes flagellin (Kofoed and Vance, 2011; Lightfield et al., 2008), the main protein component of the bacterial flagellum. NAIPs co-oligomerize with a downstream adapter protein called NLRC4. NLRC4 mediates the recruitment and activation of caspase-1 protease following NAIP activation (Mariathasan et al., 2006; Vance, 2015). The evidence that NLRC4 activation might be controlled by a ligand emerged from the observation of NLRC4-deficient murine macrophages failed to trigger caspase-1 activation in response to *Salmonella typhimurium* (Duncan and Canna, 2018; Place et al., 2021). NLRP6 is expressed predominantly in the intestine and liver, plays important roles in sensing and initiating the anti-bacterial and anti-viral immune response (Li et al., 2022a). During *Staphylococcus aureus* infection, NLRP6 is upregulated, which facilitates the assembly of the NLRP6 inflammasome complex by recruiting apoptosis associated speck-like protein containing a CARD (ASC) and caspase-1 (Ghimire et al., 2020). The NLRP9b inflammasome functions exclusively in intestinal epithelial cells (IECs) and limits rotavirus infection (Zhu et al., 2017).

NLRs are versatile cytosolic sensors that play crucial roles in detecting intracellular infections, assembling inflammasomes, and mediating immune responses against a diverse range of pathogens.

## RIG-I-Like Receptors (RLRs)

Retinoic acid-inducible gene I (RIG-I)-like receptors (RLRs) play a crucial role in detecting viral infections, driving the expression of type I IFNs and other genes that contribute together for the antiviral defense mechanism of the host (Rehwinkel and Gack, 2020). RLRs are found in many cell types and are predominantly located in the cytosol, although recent studies have indicated that RIG-I can also be present in the cell nucleus (Li et al., 2014; Liu et al., 2018). RLRs include three proteins: RIG-I, melanoma differentiation-associated protein 5 (MDA5), and laboratory of genetics and physiology 2 (LGP2). RIG-I and MDA5 are activated by immunostimulatory RNA, such as viral RNAs (Rehwinkel and Gack, 2020).

The functions of RLRs in detecting RNA viruses have been elucidated through studies on mice deficient for each respective RLR (Kato et al., 2005, 2006). RIG-I and MDA5 deficient mice showed increased susceptibility to RNA virus infections, indicating that RIG-I and MDA5 mediated antiviral responses are crucial for the elimination of RNA viruses (Song et al., 2022). RIG-I plays a critical role in recognizing various ssRNA viruses, such as paramyxoviruses (Ikegame et al., 2010), influenza A virus (IAV) (Kato et al., 2006; Lee et al., 2018), vesicular stomatitis virus (VSV) and Japanese encephalitis virus (JEV) (Chang et al., 2006). MDA5 is necessary for detecting other RNA viruses, including picornaviruses such as encephalomyocarditis virus (EMCV) (Gitlin et al., 2006; Kato et al., 2006), coxsackievirus B3 (CVB3) (Wang et al., 2010), murine norovirus (McCartney et al., 2008), Mengo virus (Kawai and Akira, 2009).

RIG-I and MDA5, upon activation, initiate downstream signaling by interacting with mitochondrial antiviral signaling (MAVs) (Goubau et al., 2013). MAVS activates its downstream components, including kinases TBK1/IKKε and the IKK complex (Fang et al., 2017). Once activated, interferon regulatory factor 3 (IRF3) and NF-κB move from the cytosol to the nucleus, promoting the transcription of various innate immune response genes, such as IFNs, antiviral genes, and pro-inflammatory genes that play a key role in coordinating the body’s innate immune response to infection. IFNs then trigger the expression of hundreds of interferon-stimulated genes (ISGs), whose products exhibit antiviral, immunomodulatory, cell growth regulatory, and metabolic regulatory functions that create an antiviral state (Chiang et al., 2014). If this reaction is effective, this response significantly limits viral replication and the spread of infection between cells. However, many, if not all, pathogenic viruses have mechanisms to escape the innate immune response, enabling them to cause disease (Kell and Gale, 2015). In addition, recent studies have shown that RLRs can recognize endogenous RNA under certain conditions, which may lead to aberrant activation of immune responses and contribute to the pathogenesis of autoimmune diseases, such as Alcardi-Goutières syndrome (AGS) and systemic lupus erythematosus (SLE) (Rehwinkel and Gack, 2020; Zhao et al., 2018).

## Other Cytosolic Innate Immune Sensor

AIM2 recognizes cytosolic DNA, an indication of pathogen invasion, through its oligonucleotide/oligosaccharide binding domain. In response to cytosolic dsDNA, AIM2 forms a macromolecular structure known as inflammasome (Rathinam et al., 2010; Sharma et al., 2019). AIM2 interacts with ASC through its pyrin domain, leading to the activation of caspase-1 (Fernandes-Alnemri et al., 2009; Kumari et al., 2020). The detection of dsDNA by AIM2 in the cytosol is crucial for initiating protection against invading pathogens such as bacteria, virus, fungi and parasites (Sharma et al., 2019). Viruses like Mouse cytomegalovirus (MCVM), vaccinia virus (VACV), and human papillomavirus (HPV) have been found to trigger the activation of the AIM2 inflammasome (Hornung et al., 2009; Man et al., 2015; Reinholz et al., 2013).

Z-DNA-binding protein 1 (ZBP1) is a Z-form nucleic acid (Z-NA) sensor that contains two Zα domains that recognize Z-DNA and Z-RNA, playing a role in defending the host against certain viruses such as influenza A virus (IAV) (Kuriakose et al., 2016; Oh et al., 2023, 2025; Oh and Lee, 2023) and herpes simplex virus 1 (HSV-1) (Lee et al., 2021b) by detecting viral nucleic acids (Jiao et al., 2020a; Karki et al., 2022; Maelfait et al., 2017; Sridharan et al., 2017; Thapa et al., 2016). The recognition of viral and endogenous Z-NA by the Zα domain of ZBP1 enables interaction with RIPK3 through Receptor-interacting protein kinase Homotypic Interaction Motif (RHIM)-RHIM homotypic interactions, which leading to inflammatory cell death (Devos et al., 2020; Jiao et al., 2020a; Karki et al., 2021a; Kesavardhana et al., 2020; Kuriakose et al., 2016).

AIM2 and ZBP1 are key cytosolic sensors that detect intracellular DNA and Z-NA, respectively, triggering inflammasome formation or inflammatory cell death to protect against invading pathogens such as bacteria, viruses.

## Importance of Cytosolic Innate Immune Sensors in Infectious, Inflammatory, and Metabolic Diseases

Innate immune sensors play a crucial role in modulating infectious, inflammatory and metabolic disease (Kanneganti, 2020; Karki and Kanneganti, 2021; Kwak et al., 2025). Numerous pathogens experience intracellular phase during infection, after the invasion, these pathogens exploit and hijack the host’s cellular environment and resources to facilitate their replication and proliferation. During this time, pathogen components, including nucleic acids and polysaccharides can be exposed to the innate immune sensors recruiting cell death molecules. Thus, cytosolic innate immune sensors are crucial for recognizing intracellular PAMPs and inducing inflammatory cell death (Bruns et al., 2014).

Among these sensors, ZBP1 typically acts as a key defense mechanism against viral infections. However, in the case of COVID-19 caused by SARS-CoV2 infection, ZBP1 contributes to cell death, cytokine storm and lethality in COVID-19 (Karki et al., 2022). In a typical viral infection, viral RNA is detected by various PRRs, such as TLRs, NLRs, and RLRs for the production of proinflammatory cytokines to initiate an antiviral response (Lee et al., 2020). HSV-1, a dsDNA virus responsible lifelong incurable, recurrent pathologies, and *Francisella*, a Gram-negative bacterium capable of causing rapid lethality, are two diverse pathogens known to activate the AIM2 inflammasome and induce cell death (Lee et al., 2021b).

While AIM2 plays a protective role in promoting host defense responses, its inappropriate activation is associated with worsening of diseases such as atherosclerosis (Fidler et al., 2021), melanoma (Fukuda et al., 2021), ischemic stroke (Denes et al., 2015; Kim et al., 2020), and post-stroke immunosuppression (Roth et al., 2021). The NLRP3 inflammasome has been identified as a trigger of Alzheimer’s disease (AD) pathogenesis. In patients with AD, both the mRNA and protein levels of NLRP3 are elevated in monocytes (Koh et al., 2021; Lee et al., 2021c). Additionally, exogenous-aggregated tau triggers the activation of the NLRP3 inflammasome in the microglia (Stancu et al., 2019).

RLRs are also involved in the innate immune response to infections caused by viruses such as SARS-CoV-2. When SARS-CoV-2 infects pneumocytes, cytosolic MDA5 and LGP2 mediate the delayed induction of interferons, which in turn establish an antiviral environment by activating ISGs. Additional evidence indicates that SARS-CoV-2 intermediates specifically induce interferon production via the MDA5 signaling pathway (Yin et al., 2021).

Cytosolic innate immune sensors are essential for pathogen defense, but their dysregulation can drive inflammatory diseases, emphasizing the need for precise regulation of immune responses.

## Virus Targeting Therapeutic Strategies

In viral infections, therapeutic strategies can be broadly classified into two categories: targeting virus itself and targeting the host. Each approach has its advantages and challenges, and both are essential in the development of antiviral therapies.

This section focuses on virus targeting therapies aim to directly inhibit key stages of the viral life cycle, blocking the virus from replicating and spreading. Maraviroc is a chemokine receptor type 5 (CCR5) antagonist that blocks the entry of HIV into host cells by preventing the virus from binding to the CCR5 receptor, which is essential for HIV entry (Woollard and Kanmogne, 2015). Replication inhibitors block viral genome replication by targeting enzymes like polymerases. For instance, Remdesivir inhibits the RNA-dependent RNA polymerase of SARS-CoV-2 (Bakheit et al., 2023; Grundeis et al., 2023). Protease inhibitors interfere with the processing of viral proteins needed for maturation. Ritonavir is commonly used in HIV treatment to inhibit viral protease activity (Hsu et al., 1998). Oseltamivir (Tamiflu) functions as neuraminidase inhibitor that blocks the release of newly formed influenza virus particles from infected cells, thereby limiting viral spread and reducing the severity of infection (Świerczyńska et al., 2022). However, one of the major challenges with virus targeting therapies is the rapid mutation rate of RNA viruses, such as HIV, influenza, and SARS-CoV-2, which can lead to the development of resistant viral strains (Badia et al., 2022; Iketani and Ho, 2024).

Virus-targeting therapies offer the advantage of directly disrupting the viral life cycle, leading to rapid control of infection, but their efficacy can be limited by the high mutation rates of RNA viruses.

## Host Targeting Therapeutic Strategies

Host targeting therapies can be classified into cytokine-based therapies and approaches that modulate innate immune sensing (Table 1). Since cytokines play pivotal roles in many immune-mediated diseases, they have been extensively studied as potential therapeutic targets (Hafler, 2007). Cytokine treatment can be used to modulate immune responses, type I IFNs are used to treat viral diseases such as hepatitis (Rasenack et al., 2003). In COVID-19, IL-1 and IL-6 antagonists have been shown to be beneficial in patients (van de Veerdonk et al., 2022). Also, patients with COVID-19 exhibit elevated levels of inflammatory cytokines, and the synergistic action of tumor necrosis factor-α (TNF-α) and IFN-γ has been shown to specifically induce cell death (Malireddi et al., 2021). Treatment with neutralizing antibodies against TNF-α and IFN-γ fully protected mice from death during cytokine storm (Karki et al., 2021b), suggesting that cytokines can boost or suppress immune responses depending on the therapeutic goal. However, cytokine therapy can sometimes exhibit high toxicity, highlighting the need for alternative approaches to modulate immune responses more precisely (Baldo, 2014).

Therapeutic strategies that directly modulate innate immune sensors rely on agonists, inhibitors, and antagonists to achieve their effects. Agonists enhance the activation of immune sensors to strengthen antiviral immunity. Inhibitors suppress overactivation to prevent inflammation-related damage, and antagonists block receptor ligand interactions.

Imiquimod is the first drug targeted for TLRs and acts as an agonist for the TLR7 receptor. It can induce the production of IFN-α, IL-6, and TNF-α, thereby modulating immunity and aiding in the treatment of tumors (Hemmi et al., 2002; Wang et al., 2005). IMO-2055 is a TLR9 agonist that may boost the efficacy of antitumor therapies by stimulating the immune response (Smith et al., 2014). CLI-095 is a small molecule inhibitor of TLR4 signaling. CLI-095 binds to cysteine 747 in the intracellular domain of TLR4, blocking MyD88-dependent and TRIF-dependent pathways activated by LPS (Kawamoto et al., 2008). Inhibition of TLR4 through CLI-095 prevents the development of autoimmune diabetes in non-obese diabetic (NOD) mice (Alibashe-Ahmed et al., 2019). Also, TLR4 is thought to play a key role in the occurrence and development of atherosclerosis, CLI-095 significantly reduces the development of atherosclerosis (Wang et al., 2016). Extracellular LPS is detected by TLR4, initiating a transcriptional response, while cytosolic LPS binds and activates non-canonical inflammasome. oxPAPC competes with LPS for binding, and directly interacts with caspase-4, and caspase-11, inhibiting LPS-induced pyroptosis, IL-1β release and septic shock (Zanoni et al., 2016). Therefore, oxPAPC and its derivatives can be potential as therapeutic agents targeting non-canonical inflammasome during Gram-negative bacterial sepsis (Chu et al., 2018). TL2-CL9 preferentially inhibits TLR2/1 signaling in primary murine macrophages (Mistry et al., 2015).

In recent years, research on MCC950 has grown, with its targets increasingly being elucidated, and its metabolism and toxicity have been a key of study (Li et al., 2022b). The NLRP3 inflammasome, which is activated by exogenous aggregated tau in Alzheimer’s disease (Koh et al., 2021), can be inhibited by the NLRP3 inhibitor MCC950, which suppresses exogenously seeded tau pathology (Stancu et al., 2019). Dapansutrile, another NLRP3 inhibitor, has been found to be safe for oral use in humans (Marchetti et al., 2018a; Sánchez-Fernández et al., 2019). ADS032 is the first described dual inflammasome inhibitor and a potential therapeutic to treat both NLRP1 and NLRP3 associated inflammatory diseases. ADS032 is an effective NLRP1 and NLRP3 antagonist in human macrophages and epithelial cells. ADS032 inhibits NLRP3 in vivo and alleviates pulmonary inflammation associated with acute silicosis. ADS032 is an effective treatment for reducing IAV-induced pulmonary inflammation and disease severity, and it also serves as a novel tool for studying the role of NLRP1 in human disease (Docherty et al., 2023). Inflammation triggered by DNA sensors plays a crucial role in disease pathogenesis.

The 4- sulfonic calixarenes inhibited AIM2-dependent T cell death following stroke, providing proof concept that they could be effective at combating post-stroke immunosuppression (Green et al., 2023). ODN A151 can inhibit AIM2 inflammasome assembly, block caspase-1 activation, and prevent IL-1β maturation in antigen-presenting cells (Kaminski et al., 2013).

Receptor-interacting protein kinase 3 (RIPK3) inhibitors present a potential target. One group demonstrate that a newly developed RIPK3 inhibitor, UH15-38, effectively and selectively inhibited IAV-induced necroptosis in alveolar epithelial cells in vivo (Gautam et al., 2024). Z-IETF-fmk, the caspase-8 inhibitor can trigger the production of proinflammatory cytokines and neutrophil influx without inducing cell death, and it protects mice against high-dose endotoxin shock (Lentini et al., 2023). The small molecule inhibitor VX-765 was shown to inhibit caspase-1 in human microglia and oligodendrocytes (ODCs) (McKenzie et al., 2018).

These advancements highlight the potential of host-targeting therapeutic strategies to modulate immune responses with precision, offering promising avenues for treating inflammatory and infectious diseases.

## Conclusion

Innate immune sensors such as TLRs, NLRs, RLRs, AIM2, and ZBP1 are pivotal components of the innate immune system, essential for detecting intracellular pathogens and initiating immune responses. These receptors trigger critical pathways that lead to the production of cytokines and interferons, which are vital for controlling infections and shaping adaptive immunity.

Targeting innate immune sensors presents a promising therapeutic strategy for treating infectious and inflammatory diseases. The development of specific inhibitors shows the potential to modulate innate immune sensor activity to manage disease. Nevertheless, further research is needed to fully understand the range of ligands recognized innate immune sensors and the intricacies of their signaling mechanisms. Advancing our knowledge in this area will facilitate the development of novel therapies targeting innate immune sensors, offering new hope for effective treatments.

In summary, targeting innate immune sensors have significant therapeutic potential, and ongoing research will be crucial in realizing this potential to combat a variety of diseases.

## Figures and Tables

**Table 1.** List of existing Innate immune sensor modulators

Therapeutic molecule	Target	Disease	Effect	Outcome	Reference
Imiquimod	TLR7	Tumor	Agonist	Induce the production of cytokine	Hemmi et al. (2002), Wang et al. (2005)
IMO-2055	TLR9	Tumor	Agonist	Enhance antitumor efficacy	Smith et al. (2014)
CLI-095	TLR4	Atherosclerosis	Inhibitor	Suppress LPS induced inflammation	Alibashe-Ahmed et al. (2019), Kawamoto et al. (2008), Wang et al. (2016)
OXPAPC	TLR2, TLR4	Sepsis shock	Inhibitor	Inhibits non-canonical pyroptosis	Chu et al. (2018)
TL2-C29	TLR2	Hepatitis C virus	Inhibitor	Inhibitor of TLR2/1 signaling	Mistry et al. (2015), Oliveira-Nascimento et al. (2012)
MCC950	NLRP3	Inflammatory diseases (atherosclerosis, myocardial fibrosis, spinal cord injury, neurological disorders, intestinal inflammation)	Inhibitor	Alleviates symptoms of associated inflammatory conditions	Coll et al. (2022), Dempsey et al. (2017), Gao et al. (2019), Jiao et al. (2020b), Zeng et al. (2021)
3,4-Methylenedioxy-β-nitrostyrene	NLRP3	Renal ischemia	Inhibitor	Protects from renal ischemia	Uysal et al. (2022)
Dapansutrile	NLRP3	Autoimmune encephalomyelitis, acute arthritis	Inhibitor	Atternuates clinical signs and improves prognosis	Klück et al. (2020), Marchetti et al. (2018a, 2018b), Sánchez-Fernández et al. (2019)
ADS032	NLRP1, NLRP3	IAV-induced pulmonary inflammation and disease severity	Inhibitor	reduces acute silicosis-associated pulmonary inflammation	Docherty et al. (2023)
4-Sulfonic calixarenes	AIM2	Post-stroke immunosuppression	Inhibitor	AIM2-dependent post-stroke T cell death inhibition	Green et al. (2023)
ODN TTAGGG	AIM2	MCMV and L. monocytogenes	Antagonist	Blocks AIM2 inflammasome activation in response to cytosolic dsDNA	Eichholz et al. (2016), Kaminski et al. (2013)
UH15-38	RIPK3	Blocked IAV-triggered necroptosis in alveolar epithelial cells in vivo	Inhibitor	UH15-38 ameliorated lung inflammation and prevented mortality	Gautam et al. (2024)
z-IETF-fmk	Caspase8	Lethal bacterial peritonitis and pneumonia	Inhibitor	z-IETD-fmk induces pro-inflammatory cytokine productin in neutrophils but not in macrophages	Lentini et al. (2023)
VX-765	Caspase1	CNS disease	Inhibitor	Reduces CNS inflammation, prevents axonal injury, improves neurobehavioral in EAE	McKenzie et al. (2018)
